# Miracle friends and miracle money in California: a mixed-methods experiment of social support and guaranteed income for people experiencing homelessness

**DOI:** 10.1186/s13063-024-08109-6

**Published:** 2024-04-29

**Authors:** Benjamin F. Henwood, Bo-Kyung Elizabeth Kim, Amy Stein, Gisele Corletto, Himal Suthar, Kevin F. Adler, Madeline Mazzocchi, Julia Ip, Deborah K. Padgett

**Affiliations:** 1 Suzanne Dworak-Peck School of Social Work, University of Southern California, 669 W. 34th Street, Montgomery Ross Fisher Building, Los Angeles, CA 90089 USA; 2 Department of Population and Public Health Sciences, Keck School of Medicine, University of Southern California, Los Angeles, CA USA; 3 Miracle Messages, San Francisco, USA; 4 Silver School of Social Work, New York University, New York, USA

**Keywords:** Homelessness, Guaranteed income, Basic income, Cash transfers, Social support, Community integration, Loneliness, Isolation, Relational poverty, Randomized controlled trial

## Abstract

**Background:**

This paper describes the protocols for a randomized controlled trial using a parallel-group trial design that includes an intervention designed to address social isolation and loneliness among people experiencing homelessness known as Miracle Friends and an intervention that combines Miracles Friends with an economic poverty-reduction intervention known as Miracle Money. Miracle Friends pairs an unhoused person with a volunteer “phone buddy.” Miracle Money provides guaranteed basic income of $750 per month for 1 year to Miracle Friends participants. The study will examine whether either intervention reduces social isolation or homelessness compared to a waitlist control group.

**Methods:**

Unhoused individuals who expressed interest in the Miracle Friends program were randomized to either receive the intervention or be placed on a waitlist for Miracle Friends. Among those randomized to receive the Miracle Friends intervention, randomization also determined whether they would be offered Miracle Money. The possibility of receiving basic income was only disclosed to study participants if they were randomly selected and participated in the Miracle Friends program. All study participants, regardless of assignment, were surveyed every 3 months for 15 months.

**Results:**

Of 760 unhoused individuals enrolled in the study, 256 were randomized to receive Miracle Friends, 267 were randomized to receive Miracle Money, and 237 were randomized to the waitlist control group. In the two intervention groups, 360 of 523 unhoused individuals were initially matched to a phone buddy. Of the 191 study participants in the Miracle Money group who had been initially matched to a volunteer phone buddy, 103 were deemed to be participating in the program and began receiving monthly income.

**Discussion:**

This randomized controlled trial will determine whether innovative interventions involving volunteer phone support and basic income reduce social isolation and improve housing outcomes for people experiencing homelessness. Although we enrolled unhoused individuals who initially expressed interest in the Miracle Friends program, the study team could not reach approximately 30% of individuals referred to the study. This may reflect the general lack of stability in the lives of people who are unhoused or limitations in the appeal of such a program to some portion of the unhoused population.

**Trial registration:**

ClinicalTrials.gov NCT05408884 (first submitted on May 26, 2022).

**Supplementary Information:**

The online version contains supplementary material available at 10.1186/s13063-024-08109-6.

## Background

People experiencing homelessness (PEH) suffer from extreme health disparities including high rates of disease (e.g., obesity, cancer, and depression) [1–7], early onset of geriatric conditions, and decades-early mortality [8–14]. More apparent drivers of these health disparities that are a direct result of homelessness include difficulty accessing quality health care and maintaining medication adherence [15–18], increased exposure to victimization [19], and accidents and extreme weather [20, 21]. Less often discussed is the role of social exclusion, isolation, and loneliness, despite the high prevalence of these factors among PEH [22, 23] and significant evidence linking them to increased morbidity and mortality [1]. This is particularly noteworthy because evidence-based interventions, most notably Housing First, can effectively address homelessness but do not necessarily increase community integration or reduce social isolation and loneliness [24–27]. This may account for why research on supportive housing programs that successfully end homelessness has yet to document a significant reduction in health disparities [28].

Given that research has established a clear causal link between social isolation and loneliness and health outcomes [29], interventions that focus on social connectedness could be considered to reduce health disparities among PEH [30]. Unfortunately, although there have been various efforts to design interventions to address social isolation and loneliness more generally, there is limited evidence of their effectiveness using rigorous designs such as randomized controlled trials [31, 32]. Of course, it is unlikely that focusing only on social isolation and loneliness without addressing other social determinants of health will significantly improve health outcomes of PEH [33]. In short, interventions that address both economic and social poverty (i.e., loneliness and social isolation) are likely needed to reduce health disparities among PEH.

This paper describes the protocols for a randomized controlled trial of an intervention initially designed to address social isolation and loneliness among PEH and subsequently paired with an economic poverty-reduction intervention. The program that delivers these interventions is a nonprofit organization known as Miracle Messages, whose mission is to “end relational poverty on the streets and, in the process, inspire people everywhere to embrace their homeless neighbors not as problems to be solved, but as people to be loved” [34]. One intervention that this program implements is known as Miracle Friends, in which an unhoused person is paired with a volunteer “phone buddy” who provides social support. Miracle Friends is not intended to replace other social services and volunteers are not expected to have any specific training to provide counseling or case management services. Instead, the goal is for volunteers to develop an informal friendship that provides the unhoused person with someone to talk to.

Another intervention that the program has recently added is known as Miracle Money, in which unhoused individuals who are participating in the Miracle Friends receive guaranteed basic income. Guaranteed basic income, sometimes referred to as a cash transfer program, has been increasingly adopted globally as a means to reduce poverty and has been shown to improve outcomes including education, employment, and health [35]. During the coronavirus pandemic, the U.S. federal government provided an unprecedented form of basic income to many Americans by funding more than $476 million in cash transfer payments [36, 37]. Although basic income programs can vary in terms of program design (e.g., who qualifies, how funds are distributed, amount and frequency of income distribution), pilot programs in the United States have mainly targeted safety-net populations in large cities including Los Angeles, Chicago, New York, Philadelphia, and Washington, DC [38]. Although many of these cities have large homeless populations [39], few basic income programs have been designed for PEH [40]. Some of the measures for the current study were borrowed from an ongoing randomized controlled trial of a 12-month program providing unconditional cash transfers to unhoused people living Colorado known as the Denver Basic Income Project [41, 42].

### Interventions: miracle friends and miracle money

In 2020, a Miracle Money proof-of-concept pilot was conducted in which nine PEH who were participating in Miracle Friends received $500 a month for 6 months. At the end of the pilot, six participants had secured housing and most reported improved social connections and less psychological distress [41]. Although participants in the pilot reported that most of their funds were used on food (30.6%) and rent (29.9%), they also reported spending funds on individualized needs that could not have been predicted, such as getting a service dog to help with anxiety, obtaining clean clothes to wear at a mosque, supporting family members, and donating to charity [41]. Importantly, many participants reported that the Miracle Friends phone buddy program was a critical component of the success or improvement that they experienced from receiving basic income [41].

To better understand the impact of the Miracle Friends intervention and how adding basic income influences outcomes, the Miracle Messages nonprofit and the University of Southern California Suzanne Dworak-Peck School of Social Work established a community–academic partnership in 2022 to design and conduct a randomized controlled trial of these interventions. Through private philanthropy, funding was secured to provide basic income to as many of 105 individuals for 1 year. The study takes place in the Los Angeles and San Francisco Bay areas of California, which have high rates of homelessness. California accounts for 30% of all people in the USA experiencing homelessness, including half (*n* = 115,491) of all unsheltered people, many of whom reside in the Los Angeles and San Francisco Bay areas [39]. In these urban regions and nationally, African Americans are overrepresented in the homeless population. Nationally, African Americans comprise 40% of the homeless population while representing only 13% of the general population [39]. This is an important factor to consider for this study, because although Whites are more likely to become homeless due to individual risk factors such as health impairments or disabilities, research suggests that African Americans are more likely to become homeless due to lack of income and social capital [43], both of which are target outcomes for the Miracle Money intervention [44]. However, it is unclear whether increased income and social support can overcome other forms of discrimination experienced by African Americans that may be required to exit homelessness [45].

The overarching research questions that this study seeks to answer are: (1) Is the Miracle Friends intervention associated with reduced social isolation and increased social support? (2) Is the Miracle Friends intervention associated with improved housing outcomes? (3) Does the addition of basic income through the Miracle Money intervention improve these outcomes of interest? (4) Are there differences in outcomes based on race?

## Methods

### Design overview

**Fig. 1:**
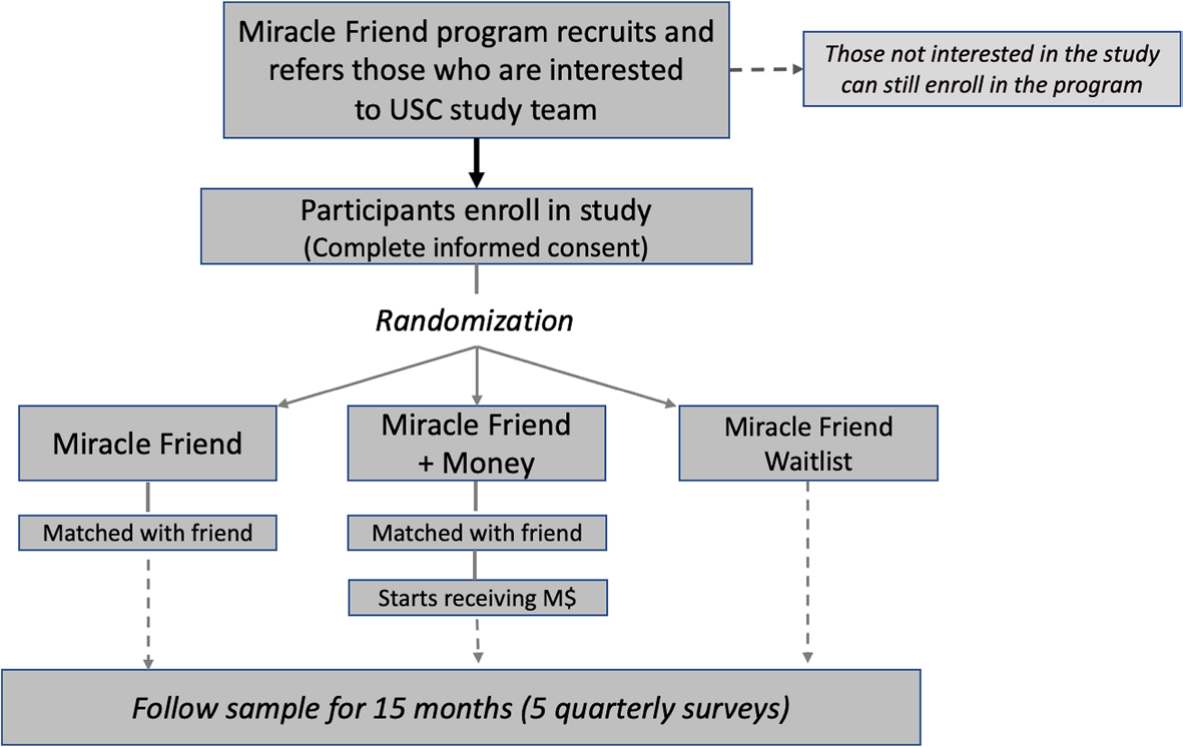
Randomized controlled trial design

Miracle Messages, the nonprofit that delivers the Miracle Friends and Miracle Money interventions, is headquartered in the San Francisco Bay Area but expanded to have staff members in Los Angeles for this study. Through partnerships with homeless service agencies or direct street outreach, Miracle Messages staff members engaged unhoused individuals to explain the Miracle Friends intervention, which requires having a phone, and signed up anyone who expressed interest in a phone buddy. Those who signed up also learned about a study to evaluate the Miracle Friends intervention, and those who expressed interest were referred to a study team affiliated with the University of Southern California. For those who agreed and provided written informed consent to participate in the study, a random number generator was used to determine sequentially whether a study participant would be offered the Miracle Friends intervention or put on a waitlist. Participants were told that they had a two-thirds chance to be offered Miracle Friends but were not told that approximately half of those offered Miracle Friends would be assigned to a third arm of the study that could receive basic income. For this group, receiving basic income through Miracle Money was contingent on participating in Miracle Friends. Figure 1 depicts how participants were randomized using a parallel-group trial design to one of three groups with a 1:1:1 allocation ratio: (a) those offered Miracle Friends only; (b) those who would be offered Miracle Money if they participated in Miracle Friends; and (c) those on a waitlist for Miracle Friends. Recruitment continued until at least 100 people began receiving Miracle Money.

Study team members interacting with participants for recruitment purposes were informed by a single study administrator via telephone just prior to enrollment about whether a participant had been randomized to receive Miracle Friends or put on a waitlist, but were blinded to whether the Miracle Friend assignment was part of the Miracle Money condition. Once enrolled, the single study administrator provided the Miracle Messages staff with the group assignment of each participant to determine whether Miracle Friends should be offered. For those assigned to Miracle Money, the possibility of receiving guaranteed income was only disclosed after it had been determined that they were participating in the Miracle Friends intervention.

Nondisclosure of the Miracle Money condition, a form of deception, was deemed necessary to avoid (a) unduly influencing people to participate in the research and (b) biasing results such that people might have engaged in the Miracle Friends program only because they were interested in guaranteed income. Because revealing that a participant may have been eligible for but was not offered basic income could potentially cause more distress than the deception, the study team will not debrief participants at the conclusion of the study about the complete study design, which is consistent with the Code of Federal Regulations on the protection of human subjects [46].

**Fig. 2:**
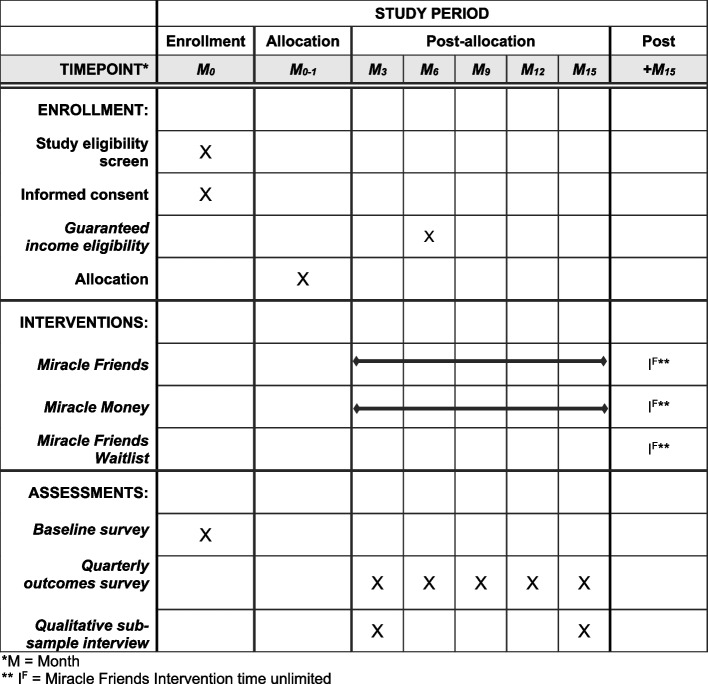
Schedule of enrollment, interventions, and assessments

Regardless of group assignment, all participants identified by a unique study identification number to preserve confidentiality have been asked to complete a baseline survey and five subsequent quarterly surveys; participants will receive a $30 gift card incentive for each completed survey. Qualitative interviews with a subset of 20 participants receiving basic income have and will be conducted shortly after receipt of the first of 12 monthly payments, with another follow-up qualitative interview scheduled around the time of the last payment. A single qualitative interview will be conducted with 20 volunteers serving as a phone buddy for at least 6 months to understand their experience of delivering the Miracle Friends intervention. Participants will receive a $30 incentive at the end of each qualitative interview. This human subjects’ research is being performed in accordance with the Declaration of Helsinki with protocols approved by the first author’s institutional review board. Reporting of study protocols follows the SPIRIT guidelines [47], which include a schedule of enrollment, interventions, and assessments, as depicted in Fig. 2. The SPIRIT checklist for Trails can be found in the Supplementary materials.

### Interventions

#### Miracle friends

Miracle Messages program staff members have recruited volunteers who want to serve as a phone buddy and unhoused individuals who expressed an interest in being matched with a phone buddy. Recruitment of volunteers has occurred primarily through people who learned about the program through media coverage, word of mouth, social media, or internet searches about helping PEH, with most volunteers signing up on the Miracle Messages website. Volunteers are required to complete an application listing any preferences for a friend (e.g., gender, language, shared interests, text or calls preferred) and attend a 30-min training call offered once a week synchronously. A recording of the training call is available as needed to those with significant scheduling conflicts. Volunteers receive a program handbook that outlines expectations for logging into an online platform to record any contact or attempted contact with an unhoused friend. After completing a waiver of liability, volunteers receive a phone number through Dialpad or similar service (which allows the volunteers to avoid divulging their personal phone numbers if they wish) and are subsequently matched with an unhoused friend, usually in a few weeks. Weekly support calls are offered to all volunteers, in addition to one-on-one support provided upon request based on completing the contact logs. Currently, there are approximately 300 Miracle Friend volunteers.

Recruitment of unhoused individuals has happened primarily through (a) site visits at local partner sites, including shelters, transitional housing facilities (e.g., tiny homes), converted hotels or motels for unhoused individuals, etc.; (b) referrals from caseworkers and social workers at these partner sites; and (c) limited direct outreach on the streets in and around partner sites or at events designed to address the needs of PEH (e.g., food pantries, homeless connect events). Miracle Friends program staff members explain the intervention and sign up anyone who expresses an interest in a phone buddy. Like with the volunteers, unhoused participants are also asked to complete an application listing any preferences for a friend (e.g., gender, language, shared interests, text or calls preferred).

Matching of unhoused individuals and volunteers has been primarily based on preferences and shared interests, as indicated on the enrollment application. Once a match is determined, volunteers receive the phone number of their assigned unhoused friend and are asked to make contact as soon as possible. The program has no time limit, and the development of friendships is expected to occur naturally over time and be unique to each matched pair. Communication is bidirectional in that both participants can call or text each other. Volunteers are encouraged to provide a friendly voice and be a compassionate listener without judgment; there is no expectation of formal counseling or case management. If a lack of fit occurs or communication is not maintained between the two individuals, the program offers to rematch the unhoused individual, volunteer, or both. If a volunteer or Miracle Messages staff members cannot contact an unhoused person after five attempts by the volunteer and one attempt by the staff, the person is removed from the list of people needing to be matched—effectively representing a discharge from the program. However, participants can reenroll at any time. Once matched, volunteers are expected to attempt weekly phone voice or text contact. Volunteers log their efforts on the program’s online platform after each attempt, helping the program monitor the progression of friendships and address any issues. Based on volunteer responses, the Miracle Friends program can provide referrals to the unhoused person if there is a more urgent need or contact a formal service provider if authorized by the unhoused individual.

#### Miracle money

Whereas the Miracle Money proof-of-concept provided $500 for 6 consecutive months, for this study, funds were raised so that Miracle Messages could provide up to 110 individuals with $750 per month for 12 months. This was based on pilot work that suggested that $500 per month meaningfully changed lives [41] but recognition that a living wage in these cities would be much higher [48]. As noted, Miracle Money is not advertised to those interested in the Miracle Friends intervention; unhoused individuals are only notified about the possibility of receiving basic income if they are randomized to the Miracle Money condition and participate in the Miracle Friends interventions. Although participation was defined as having had at least two contacts with a matched volunteer in a 1-month period, at the outset of the study there were efforts to ensure that the two contacts could be characterized as “meaningful” based on volunteer feedback to help ensure that the matching process would result in a prolonged friendship or relationship. Yet because many of the initial contacts could not be confirmed to be meaningful, Miracle Messages increasingly came to accept any type of contact with volunteers as meeting the criteria of Miracle Friend participation that would then allow participants to start receiving Miracle Money.

Volunteers are notified when the person with whom they have been matched becomes eligible for Miracle Money so that the volunteer can be present during the phone call in which the study participant is offered Miracle Money. Those who qualify and decide to move forward with receiving basic income payments are asked to complete an application for a cash transfer technology company known as AidKit, which has been contracted to process monthly payments sent via a debit card or direct deposit. Although we do not expect many people to turn down Miracle Money, we envision that some may decide against receiving income payments based on how it may affect other benefits that depend on income (e.g., Supplemental Security Income, Supplemental Nutrition Assistance Program), although the intent of guaranteed basic income is to supplement rather than replace existing resources. When participants are first offered Miracle Money, the Miracle Messages staff discusses benefits and encourages participants to discuss the topic with a case manager; referrals to financial coaching are also offered. Participants are told that they can use the money in any way they choose but that the program requests the money not be used for any illicit purposes.

### Recruitment and enrollment

As noted, study recruitment has occurred via the Miracle Messages program, which notifies the study team about unhoused individuals interested in participating in an evaluation of the Miracle Friends intervention. This has occurred in person when the study team accompanied Miracle Messages staff members to outreach events or through a shared online referral document that provided the study team with the contact information of individuals interested in Miracle Friends and the evaluation study. Once the study team confirmed that an individual met the inclusion criteria—which included (a) being 18 years old or older; (b) speaking English or Spanish; (c) currently experiencing homelessness based on definition from the U.S. Department of Housing and Urban Development; and (d) expressed interest in the Miracle Friends phone buddy intervention—enrollment into the study occurred in person with signed informed consent or over the phone with informed consent, requiring an electronic signature completed via email using the REDCap platform [49, 50]. The informed consent process ensures that individuals understand that participation in the study may result in being assigned to a waitlist where they would be ineligible to participate in Miracle Friends for 15 months unless they withdrew from the study. Once enrolled in the study, participants complete a baseline survey and learn whether they have been randomly assigned to the waitlist or Miracle Friends. Surveyors are blinded to whether a participant is assigned to the group that will be offered Miracle Friends only or the group that will be offered Miracle Money if they participate in Miracle Friends. The Miracle Messages administrative staff is notified to which of the three groups a participant has been assigned to determine whether someone should be matched to a phone buddy and monitor which individuals become eligible for Miracle Money based on participation in Miracle Friends. Study recruitment continued until at least 100 people started receiving Miracle Money.

### Quantitative data collection procedures

Upon enrollment in the study and regardless of group assignment, the participants complete a baseline survey that takes approximately 45 min and includes questions about demographic characteristics, homelessness history, physical and mental health status, health service utilization, employment, substance use, socioeconomic status, and income. Participants are also asked to provide an email address and collateral contact information for one or more people to ensure that the study team can reconnect with them when it is time to complete a shorter survey five more times, one every 3 months (i.e., quarterly) until 15 months. The quarterly survey asks about similar topics and takes approximately 25 min. Surveys can be completed in person or over the phone, with responses recorded by a surveyor directly into the REDCap data management platform [49, 50] that is also used to support data visualization to monitor data quality and study retention. Upon request, participants also receive a link to self-administer the survey in English or Spanish. Table 1 describes all study measures.

**Table 1:** Study measures and scales

Measure	Description
***Primary outcomes***	
Housing status	Housing status is measured using questions from the Los Angeles County Homeless Count demographic survey [51]. Participants are asked, “In the past 30 days, please indicate all places where you have slept for at least one night.” Response options include (1) My own apartment or home; (2) Someone else’s apartment or home; (3) In a shelter, emergency, temporary housing; (4) Hotel/motel provided by an agency; (5) Outside on the street, park, or beach; (6) Tent or makeshift shelter; (7) In a bus station, train station, airport; Abandoned building; (8) In a vehicle (car, van, RV, truck); (10) An institution, hospital, or facility. A follow-up question could include, “If you stayed in more than one place, where did you stay the most?” Participants are also asked up to three possible questions with “yes” or “no” as response options. These include: Do you currently have a case worker who can help you with housing assistance? Do you currently have a housing assistance voucher? Are you on a waitlist for housing assistance?
Loneliness	UCLA Loneliness Short Version is a validated instrument [52] that measures three dimensions of loneliness: relational connectedness, social connectedness, and self-perceived isolation. Participants are asked three questions: (1) How often do you feel that you lack companionship? (2) How often do you feel left out? (3) How often do you feel isolated from others? Response options include: Hardly ever; Some of the time; Often
Social support	The Oslo Social Support Scale is a 3-item self-report measure of the level of social support [53]. Participants are asked three questions with different response options. The first is: “How many people are so close to you that you can count on them if you have great personal problems?” with response options of: None; 1–2; 3–5; or 5 + . The second is: “How much interest and concern do people show in what you do?” with response options of: None; Little; Uncertain; Some, or A lot. The third is: “How easy is it to get practical help from neighbors if you should need it?” with response options of: Very difficult; Difficult; Possible; Easy; or Very easy
***Other covariates***	
Demographic and historic information (baseline only)	The baseline survey includes questions about the following demographic and historic information taken from previous research studies related to homelessness: age, sex, gender, sexual orientation, race and ethnicity, country of origin, relationship status, children, level of education, military service, health insurance, public benefits, employment, monthly income, criminal justice involvement, homeless history, housing preferences, and health conditions
Employment status	Employment status is measured using questions from the Los Angeles County Homeless Count demographic survey [51]. Participants are asked, “What is your employment status?” with the following response options: Employed, full-time (35 h a week or more); Employed, part-time; Unemployed, looking for work; Unemployed, not looking for work; Retired, receiving retirement benefits; and Retired, not receiving benefits. Participants are also asked to indicate their total monthly income
Financial well-being	Adapted questions from the Consumer Finance Protection Bureau’s abbreviated 5-item measure are used. Questions include: “Usually, do you have enough money to meet your needs?” with response options: Completely; Mostly; Moderately, A little; Not at all. “How often (always, often, sometimes, rarely, never) does this statement apply to you? (1) I have money left over at the end of the month; and (2) My finances control my life.” “How well (completely, very well, somewhat, very little, not at all) do the following statements describe you or your situation? (1) Because of my money situation, I feel like I will never have the things I want in life; (2) I am just getting by financially; and (3) I am concerned that the money I have or will save won’t last.” Participants are also asked if and what health insurance they have; monthly benefits that they receive (e.g., general relief, social security disability income or Supplemental Security Income; Supplemental Nutritional Assistance); if they have a bank account; and whether they were able to pay all bills last month
Food security	Two items taken from United States Department of Agriculture Household Food Security Survey ask about how often it is usually true for the participant (“often,” “sometimes,” “never,” or “I don’t know”) in the past 30 days: “I worry whether my food will run out before I get money to buy more” and “The food I bought just doesn’t last and I don’t have money to get more.”
Life satisfaction	General life satisfaction is assessed using a 7-item validated measure from the World Health Organization Quality of Life toolkit [54]. Questions include: In general, how satisfied are you with your life? How satisfied are you with your ability to perform your daily living activities? How satisfied are you with your personal relationships? How satisfied are you with the support you get from your friends and family? How satisfied are you with your ability to provide for or support others? How satisfied are you with the way you spend your time? How would you rate your quality of life? Response options for all questions are: Very satisfied; Satisfied; Neither satisfied nor dissatisfied; Dissatisfied; Very dissatisfied
Physical health	The PROMIS Global Health Scale Version 1.2 (2 items, self-rated physical health and activities) is used: “In general, how would you rate your physical health?” Response options: Excellent; Very good; Good; Fair; Poor. “To what extent are you able to carry out your everyday physical activities such as walking, climbing stairs, carrying groceries, or moving a chair?” Response options: Completely; Mostly, Moderately; A little; Not at all
Mental health	PROMIS is also used to assess mental health (2 items, self-rated mental health, frequency bothered by symptoms): “In general, how would you rate your mental health, including your mood and your ability to think?” Response options: Excellent; Very good; Good; Fair; Poor. “In the past 7 days, how often have you been bothered by emotional problems such as feeling anxious, depressed, or irritable?” Response options: None at all; A little; A moderate amount; Very much; An extreme amount
Social health	PROMIS is used to assess social health (1 item, self-rated satisfaction with social activities and relationships): “In general, please rate how well you carry out your usual social activities and roles (this includes activities at home, at work, and in your community, and your responsibilities as a parent, child, spouse, employee, friend, etc.).” Response options: Excellent; Very good; Good; Fair; Poor
Substance use	Participants are asked: In the last month, how often did you: (1) have a drink containing alcohol? (2) take substances like cannabis/marijuana, meth, cocaine, fentanyl, heroin, prescription opioids, etc.? Response options include: Never; once or twice; Once a week; 2 to 3 times a week; 4 to 6 times a week; Daily; Prefer not to answer. Participants are also asked if they are currently receiving treatment for substance use (including alcohol) and how much money in the past month that they have spent on (1) Cigarettes and (2) drugs and alcohol
Sleep	Sleep disturbance is measured using two questions from the WHO Quality Of Life Scale [54]: Do you have any difficulties with sleeping? How much do sleep problems worry you? Response options: None, A little, A moderate amount; Very much; An extreme amount
Psychological distress	Via the Kessler Psychological Distress Scale [55], participants are asked how often over the past month that they have had certain feelings on a scale of: None of the time; A little of the time; Some of the time; Most of the time; All of the time. Feelings include: Tired out for no good reason; Nervous; So nervous that nothing could calm you down; Hopeless; Restless or fidgety; So restless you could not sit still; Depressed; That everything was an effort; So sad that nothing could cheer you up; and Worthless
Health care utilization	Participants are asked how many times they have seen a doctor or health care provider for nonemergency care in the past 30 days
Criminal justice involvement	Participants were asked: In the past 3 months, have you had contact with the police? In the past 3 months, how many times have you been arrested? Did a new arrest lead to new charges pending against you? During the past 3 months, did you stay one or more nights in prison or jail?
Perceived discrimination	A short version of the daily discrimination subscale includes six items and attributions for why a person experienced discrimination. Items regarding discrimination include: You are treated with less courtesy than other people; You receive poorer service than other people at restaurants or stores; People act as if they think you are not smart; People act as if they are afraid of you; You are followed around in stores; You are threatened or harassed. Response options are: Often; Sometimes; Rarely; Never. Participants who have perceive discrimination are asked, “What do you think is the main reasons for these experiences?” and can check all that apply: (1) Your ancestry or national origins; (2) Your gender; (3) Your race; (4) Your religion; (5) Your weight/height; (6) Some other aspect of your physical appearance; (7) Your sexual orientation; (8) Your education or income level; (9) Your housing status

At the end of each survey, participants are asked two open-ended questions: (1) Looking back over the last 3 months, what do you think was the most significant change to your quality of life? (2) Tell me in a sentence or two, what are your most important goals in life right now? If the survey is administered by a surveyor, responses are entered verbatim as much as possible; if self-administered, participants enter their responses directly. All surveyors complete a training on best practices for trauma-informed interviewing.

### Qualitative data collection procedures

#### Miracle money recipients

Qualitative interviews will be conducted in English with a subset of 20 participants who receive basic income—shortly after receipt of the first of 12 monthly payments and again around the time of their last payment. During the first quarterly survey, participants who received monthly income will be purposively sampled and asked if they would be interested in participating in an additional in-depth qualitative interview to learn more about their experience with the program. Maximum variation sampling will be used to ensure differences in race, ethnicity, and gender in our qualitative subsample. Those who are interested and agree to participate will be contacted by a research team member who has been trained in conducting qualitative interviews. Interviewers will schedule a convenient time and place to meet unless participants request a phone interview. Once an addendum consent is completed, semistructured interviews will last between 30 and 60 min and include questions about how participants view their lives, general experiences with the Miracle Friends program, and the Miracle Money program, including how they use the money and any difference it has made in their lives. Interviews will be audio recorded and transcribed verbatim.

#### Miracle friend volunteers

A single qualitative interview will be conducted with 20 volunteers who served as a phone buddy for at least 6 months to understand their experience of Miracle Friends. Any volunteer who has been engaged in the intervention for at least 6 months will receive an email from the Miracle Messages program that informs them of the study and refers them to the study team if they are interested in speaking about their personal experience with the program. Thirty-three volunteers have indicated that they would be interested; 20 have been purposively sampled to include volunteers who had participants in the Miracle Friends program and Miracle Money program. Phone or videoconferencing interviews have been conducted using a semistructured interview guide that included questions about how and why participants became a volunteer, experiences with the program, and whether they felt that they had an impact on their unhoused friend or if their friend has affected their life. Questions about how the program could be improved were also included. Interviews have typically lasted between 25 and 35 min and were audio recorded and transcribed verbatim. Written informed consent has been waived by the institutional review board for volunteer interviews.

### Quantitative data analysis

#### Social isolation outcomes

To evaluate the efficacy of the Miracle Friends intervention on social support, psychological distress, and loneliness, we will use responses to the Oslo Support Scale [53], Kessler Psychological Distress Scale [55], and UCLA Loneliness Scale [52], respectively, at the final survey (Quarter 5). Each measure will first be summed to develop a total score, then dichotomized based on cutoffs for each measure based in the literature—i.e., poor social support will be coded as 1 if the Oslo Support Scale sum score is less than 9 (indicative of poor social support) and 0 otherwise; psychological distress will be coded as 1 if the Kessler Psychological Distress Scale sum score is greater than 25 (indicative of high psychological distress) and 0 otherwise; and loneliness will be coded as 1 if the UCLA Loneliness Scale sum score is greater than or equal to 6 (indicative of high loneliness) and 0 otherwise. Each dichotomized score will be modeled as a function of treatment group (1 = Miracle Friends or Miracle Money group members who participated in at least one phone buddy intervention, 0 = waitlist group); covariates including demographic characteristics, physical and mental health, and substance use; and a random intercept for city in random intercept logistic regression models.

#### Housing outcomes

The association of Miracle Friends with housing outcomes will be evaluated using logistic regression with a random intercept, modeling the dichotomized outcome of exited homelessness (1 = individual responded “My own apartment or home” or “Someone else’s apartment or home,” 0 = otherwise) as a function of treatment (1 = Miracle Friends group members who participated in at least one phone buddy intervention, 0 = waitlist group), with the same covariates and random intercept.

#### Differences in treatment effect based on race

To evaluate if outcomes differ by race, all models will be run with the addition of an interaction between race and treatment.

#### Statistical power

To reach a target goal of 105 participants receiving basic income through Miracle Money, recruitment efforts have yielded more than 200 individuals in each treatment group. Assuming that each arm will have at least 105 participants, our study can detect the hypothesized 50% difference in proportion of people exiting homelessness at Quarter 5 between the Miracle Money and waitlist group (assuming 60% of the Miracle Money group exits homelessness and 10% of the waitlist group exits homelessness) using a *p*-value threshold of 0.05 with power exceeding 95%. When comparing the Miracle Friends group to the waitlist group, our study can detect the hypothesized 15% difference in proportion of people exiting homelessness at Quarter 5 between the Miracle Friends and waitlist group (assuming 25% of the Miracle Money group exits homelessness and 10% of the waitlist group exits homelessness) using a *p*-value threshold of 0.05 with power exceeding 60%. Differences in proportions of the social isolation outcomes will be detected at powers identical to the housing outcome, assuming identical differences in proportions and *p*-values. Regarding the effect modification of treatment by race in exiting homelessness, our study will be able to detect a difference in proportion of groups exiting homelessness of 35% with power exceeding 20%. Main effect calculations were done on unadjusted models using the WebPower R package, and the effect modification power calculations were performed via simulation.

### Qualitative data analysis

In keeping with qualitative analytic procedures, each interview has been transcribed verbatim by the research team member who conducted the interview. Transcripts have been distributed and shared with the larger team for review. A team approach will occur in data analyses, where instruction in coding will be supplemented with test cases in which two researchers read and code a transcript and then meet to discuss discrepancies and arrive at consensus. Given the focused nature of the inquiry, the resulting codebook will be a reflection of the questions that were asked. At the same time, interviewees often have shared greater depth or alternative descriptions that will “earn their way” into the analyses and interpretation [56]. In the final stage of analysis, broader interpretation will be sought to identify recurrent themes agreed on by consensus and recorded as memos.

## Results

**Fig. 3:**
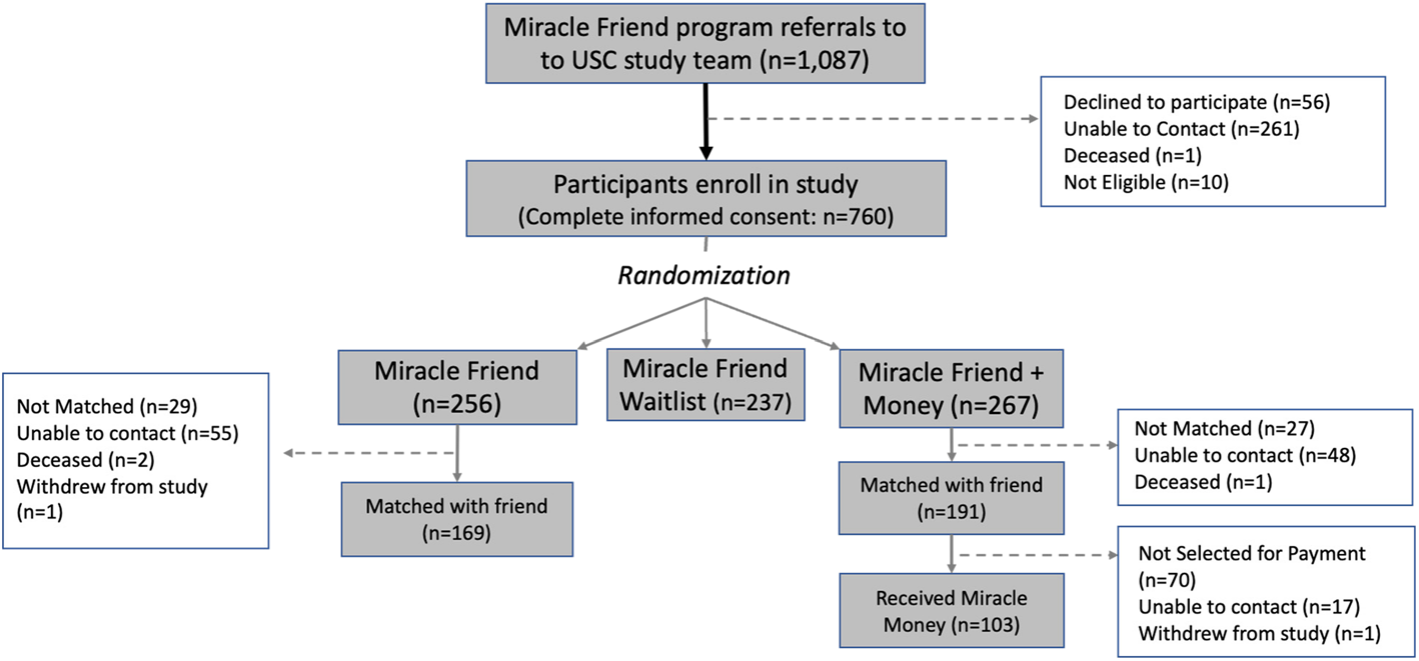
Consort diagram

Between May 2022 and July 2023, when recruitment ended, the Miracle Friends program referred 1087 unhoused individuals to the study team and 760 enrolled in the study. As depicted in Fig. 3, among those who enrolled, 256 were randomized to Miracle Friends only, 267 were randomized to Miracle Money, and 233 were randomized to the Miracle Friends waitlist control group. Thus far, 360 unhoused individuals have been matched to a phone buddy from across the two intervention groups (169 in Miracle Friends only and 191 in the Miracle Money group), with 56 people still not matched. We have been unable to contact 103 people after they were assigned to a treatment group. Of the 191 study participants in the Miracle Money group who were initially matched to a volunteer phone buddy, 103 were verified as participating in Miracle Friends and began receiving monthly income and 70 people were not selected because they were not participating in Miracle Friends. One person withdrew from the study and we were unable to contact 17 others in the Miracle Money group who had been initially matched with a Miracle Friend.

## Discussion

This randomized controlled trial was designed to evaluate the effectiveness of the Miracle Friends and Miracle Money interventions as compared a control group that received neither intervention. Although 760 unhoused individuals have enrolled in the study, the study team has been unable to contact more than 30% of the individuals who initially expressed an interest in the Miracle Friends phone buddy program and were referred to the study. This may reflect the general lack of stability in the lives of PEH or limitations in the appeal of such a program to some portion of the unhoused population. Future analysis will compare those who engaged in the intervention versus those who did not to provide some insight into potential differences. It is also unclear how increased transparency about the prospect of basic income would have changed engagement and retention in the program or study. Another factor that may have contributed to attrition is a delay in the matching process that sometimes occurred when not enough volunteers were available to meet the demand, which could have discouraged unhoused people who signed up for the program from participating. Future analyses will attempt to understand who ended up volunteering for this program and why they volunteered, as well as how long it takes to match participants and under what circumstances a match is successful.

### Trial status

This version of the study protocols (2.1) includes two amendments to the initial protocols approved by the institutional review board on April 21, 2022. Recruitment began on May 30, 2022, and ended July 10, 2023. Final enrollment into the Miracle Money intervention was delayed until August 2023 to give study participants an opportunity to meet the criterion for receiving basic income (i.e., participating in the Miracle Friends program).

### Limitations

This study is unique in that it represents the first known experiment of interventions that provide social support and guaranteed income for PEH. Challenges include study retention, given that participants have been recruited while experiencing homelessness, either sheltered or unsheltered. There may also be differential retention rates because people receiving basic income may be more likely to complete follow-up surveys as compared to the waitlist or Miracle Friends only groups. The likelihood that unhoused participants continue with the program may depend on the type of volunteer match they receive, including concordance between the two parties based on factors such as race, ethnicity, age, or gender, which this study will not examine because we have not captured dyadic information. The study also will not follow people after the basic income funding has ended. It should also be noted that this intervention primarily focused on individuals who had a cell phone, which research suggests is much of the homeless population [57].

### Conclusion

The Miracle Friends and Miracle Money programs in California offer an innovative approach to addressing the needs of PEH. This randomized controlled trial will help determine whether these programs reduce social isolation and loneliness and lead to better housing outcomes. The results will likely be of interest to policymakers, who have struggled to find appropriate system-based responses to the growing problem of homelessness in California and elsewhere. Results of the trial will be shared through peer-reviewed publications and other public dissemination efforts (e.g., policy briefs and presentations).

## Protocol version

2.1 on 3/24/24.

## Trial oversight

This study is being conducted at the Center for Homelessness, Housing, and Health Equity Research at the University of Southern California (USC) Suzanne Dworak-Peck School of Social Work and in collaboration with Miracle Messages, which is a U.S. 501(c)(3) nonprofit organization, EIN# 82–4,179,328. Throughout study recruitment, these two organizations met weekly since Miracle Messages identified potential candidates for the study and referred people to the study team at USC, which enrolled and obtained informed consent from participants. All interventions were implemented by Miracle Messages and all data collection was conducted by USC. Ethical oversight of the study was done through the USC Human Research Protection Program. USC research team members will continue to meet weekly throughout the study with oversight provided by the director of the Center for Homelessness, Housing and Health Equity Research.

## Trial results

Results of this trial will be posted on ClinicalTrials.gov and disseminated through peer-reviewed publications.

### Supplementary Information

**Supplementary Material 1.**

## Data Availability

All data from this research will be deidentified and made available in a public data repository (TBD).
